# Moxibustion Alleviates Injury in a Rat Focal Segmental Glomerulosclerosis Model

**DOI:** 10.1155/2017/7169547

**Published:** 2017-05-28

**Authors:** Yi Li, Yuxia Sun, Chunling Zhang, Ke Wang, Peicheng Shen, Di Huang, Wen Ma, Jin Zhang, Lin Li, Liqun He

**Affiliations:** ^1^Department of Nephrology, Shuguang Hospital Affiliated to Shanghai University of Traditional Chinese Medicine, Shanghai 200021, China; ^2^Department of Cardiology, Shuguang Hospital Affiliated to Shanghai University of Traditional Chinese Medicine, Shanghai 201203, China; ^3^Laboratory of Integrative Medicine Surgery, Shuguang Hospital Affiliated to Shanghai University of Traditional Chinese Medicine, Shanghai 201203, China; ^4^Department of Nephrology, Shuguang Hospital Affiliated to Shanghai University of Traditional Chinese Medicine, Traditional Chinese Medicine Institute of Kidney Disease, Shanghai University of Traditional Chinese Medicine, Shanghai Key Laboratory of Traditional Chinese Clinical Medicine, Shanghai 201203, China; ^5^Acupuncture Anesthesia Research Section, Shuguang Hospital Affiliated to Shanghai University of Traditional Chinese Medicine, Shanghai 200021, China; ^6^Shanghai University of Traditional Chinese Medicine, Shanghai 201203, China

## Abstract

**Objectives:**

To evaluate the therapeutic effects of moxibustion at Shenshu (BL-23) and Geshu (BL-17) acupoints in a focal segmental glomerulosclerosis (FSGS) model in rats.

**Methods:**

A FSGS rat model was established by single nephrectomy and repeated injection of doxorubicin. The FSGS rats were randomly divided into the model group, losartan (positive control) group, Shenshu moxibustion group, and Geshu moxibustion group. Molecular indicators of kidney function and renal pathological changes were monitored.

**Results:**

Urinary protein, serum creatinine, urea nitrogen, and serum uric acid were significantly reduced after 12-week intervention with losartan, Shenshu, or Geshu moxibustion. Renal *α*-SMA, FN, and TGF-*β* were also decreased, while podocin and nephrin protein and mRNA were increased. The pathological damage in renal tissue was obviously alleviated by all three treatments, which suggests that moxibustion may have similar efficacy to losartan in the treatment of FSGS.

**Conclusion:**

Moxibustion alleviates podocyte injury and inhibits renal interstitial fibrosis in the FSGS rat model, thereby minimizing the progression of glomerular sclerosis and improving renal function.

## 1. Introduction

Focal segmental glomerulosclerosis (FSGS) is characterized by scarring to a subset of the glomeruli of the kidney and can occur as a result of infection, drugs, or systematic diseases such as diabetes or lupus or other glomerular diseases. The main clinical manifestation of FSGS is proteinuria. The severity of the proteinuria and the response to treatment are closely associated with the kidney prognosis, and patients with nephrotic syndrome are prone to develop end stage renal disease [1–3]. Therefore, the primary goal of FSGS treatment is to reduce proteinuria in a short time to protect the kidney [4, 5].

In recent years, traditional Chinese medicines (TCMs) have been considered as effective treatments for a variety of different physical conditions, including renal diseases. Acupuncture and moxibustion are thought to contribute to disease treatment by stimulating the surface of meridian acupoints. Compared to acupuncture, moxibustion is motivated by “warm stimulation.” The main material for moxibustion therapy is moxa wool, which is used to construct cones or sticks. The moxa sticks or cones are ignited prior to application on the surface of meridian acupoints via scarring or nonscarring methods. However, rigorous, randomized controlled trials of moxibustion have not been described, which may account for the poor awareness of this therapy method.

Nevertheless, animal experiments support the potential role of both acupuncture and moxibustion in the treatment of renal diseases. Paterno et al. [6] demonstrated that electroacupuncture at Zusanli (ST-36) and Taixi (KI-3) acupoints and moxibustion at the Shenshu (BL-23) acupoint improve the serum creatinine, 24-h urinary protein, glomerulosclerosis, and tubulointerstitial fibrosis in a 5/6 nephrectomy rat model. Further study by this group [7] showed that the treatment mechanism may be related to the regulation of renal sympathetic nerve activity and nitric oxide levels and the reduction of arterial blood pressure. The potential role of nitric oxide release from nerves has also been noted in the response to local thermal stimulation on Riyue (GB-24), Weizhong (BL-40), and Chengfu (BL-36) acupoints to inhibit the physiological activities of the Oddi sphincter and the anal sphincter [8–10]. Additionally, several investigators have demonstrated that thermal stimulation within the regions of Qimen (LR-14), Neiguan (PC-6), and Yinmen (BL-37) acupoints increases the expression of heat shock protein 70 (HSP70) and elevates reactive oxygen species, which protects liver and heart muscles from ischemia-reperfusion injury [11–15]. Matsumoto-Miyazaki et al. [16] observed reduced renal vascular resistance after indirect moxibustion at the bilateral Shenshu acupoints of 43 chronic kidney disease patients. At present, however, direct reports of moxibustion therapy for proteinuria in patients with chronic glomerular disease are relatively rare. Therefore, moxibustion therapy is worthy of further in-depth investigation.

In this study, we used single nephrectomy and repeated injections of doxorubicin [17] to establish FSGS in rats. Using the angiotensin II receptor antagonist losartan as a positive control therapeutic, we evaluated the potential therapeutic effects of mild Shenshu moxibustion (BL-23) and Geshu moxibustion (BL-17). We monitored the urinary protein level; kidney function; expression of renal fibronectin (FN), *α*-SMA, TGF-*β*, podocin, and nephrin; and pathomorphology of FSGS kidney tissues of different groups of rats. Our results support the potential therapeutic value of moxibustion at Shenshu (BL-23) and Geshu (BL-17) acupoints in the treatment of FSGS.

## 2. Methods

### 2.1. Animals

Fifty-one clean-grade male Sprague Dawley rats (bodyweight of 140–160 g) were purchased from Shanghai Sippr-BK Lab Animal Co., Ltd. (Shanghai, China, animal qualification certificate number: SCXK [Shanghai] 2013–0016). All experimental rats were fed common chow (containing 21% crude protein, 4.5% crude fat, and 5% crude fibers, purchased from Shanghai Shilin Biotechnology Co., Ltd., Shanghai, China) and housed in the Animal Experimental Center of Shanghai University of TCM, with a room temperature of 22–25°C and humidity of 50%. Animal experiments and euthanization procedures were in accordance with* Guidelines on the Treatment of Experimental Animals* issued by Ministry of Science and Technology in 2006 and relevant animal ethics standards listed in* Ethical Issues in Animal Experimentation* in 2009.

### 2.2. Establishment of the FSGS Model

All rats were acclimated for a week and then were weighed and numbered by their order in weight (from light to heavy). Corresponding to numbers generated by SPSS statistical software, seven rats were randomly selected for sham operation by laparotomy to expose the kidneys and remove the renal capsule without dissecting the kidney tissue, followed by suturing layer by layer. These animals were then tail-vein injected with 0.5 ml normal saline on day 4 and day 18 after sham operation. The other 44 rats received two intravenous injections of doxorubicin two weeks apart and unilateral nephrectomy to model glomerulosclerosis. All animals were fasted with free access to water 12 h before operation and anesthetized by intraperitoneal injection of 50 mg/kg 1.5% pentobarbital sodium (Merck Drugs and Biotechnology, Germany, batch number: 20130112). After the corneal reflex had subsided, the animals were fastened on the operating table, followed by hair shaving and routine disinfection. A 1–1.5 cm dorsal incision was made to expose the left kidney; remove the fat tissues and adrenal gland around the kidney; ligate the left renal artery; and resect the left kidney. The incision was then sutured. The animals were tail-vein injected with 3 mg/kg (postoperative day 4) and 2 mg/kg (postoperative day 18) of doxorubicin, which was dissolved in sterile water at a dilution of 2 mg/ml (10 mg doxorubicin/syringe; Shenzhen Main Luck Pharmaceuticals, Inc., Guangdong Province, China; batch number: national approved medicine H44024359). The urinary protein of the viable rats was quantified seven days after the second doxorubicin injection. Animals with >100 mg/24 h urinary protein indicated successful modeling and were included in subsequent experiments, with monitoring for 12 consecutive weeks.

### 2.3. Grouping and Intervention

**Figure 1:**
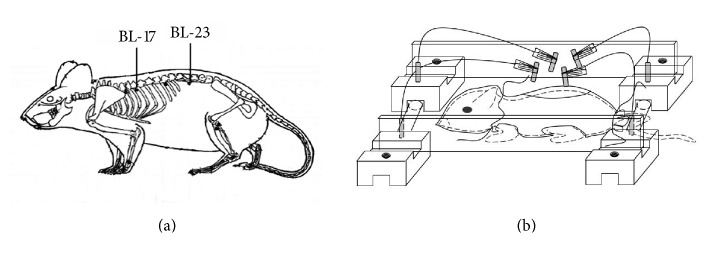
Experimental application of moxibustion in rats. (a) The body surface position of acupoints in the rat. (b) Schematic presentation of the self-made device for administration of moxibustion to rats.

Forty-four successfully modeled FSGS rats were numbered from 1 to 44 according to the quantity (from small to large quantity) of urinary protein. Corresponding to numbers generated by SPSS statistical software, the 44 rats were randomly divided into the model, losartan, Shenshu moxibustion (BL-23-MO), and Geshu moxibustion (BL-17 MO) groups (*n* = 11 rats per group). Intervention was initiated in each group on the same day. Animals in the sham-operation, model, and moxibustion groups were given 5 ml/kg distilled water by gavage once a day. Rats in the losartan group were given losartan potassium tablets, which were dissolved in distilled water at a dilution of 5 mg/ml immediately before the administration, by gavage once a day (100 mg/tablet, produced by Hangzhou MSD Pharmaceutical Co., Ltd., Hangzhou, China; batch number: national approved medicine H20030654). Rats in the Shenshu and the Geshu moxibustion groups received mild moxibustion at Shenshu (bilateral) acupoints or Geshu (bilateral) acupoints, every other day (30 min per acupoint).  Figure 1(a) shows the locations of the two acupoints. To localize the bilateral Shenshu (BL-23) acupoints, rats were fixed in a prone position with the two hind limbs straightened. The highest points on both sides of the iliac and lumbar spinous process were located and crossed with the horizontal connection of the spine, which was 1.5 inches away from the 2nd upper spinous process position based on the middle toe-identical unit of the rat body in the hind limb. To localize the bilateral Geshu acupoints, the Dazhui acupoint (GV14, at the center of the back between the 7th cervical vertebra and first thoracic spine) was first located, and the position 1.5 inches away from the 7th thoracic spinous process based on the middle toe-identical unit of the rat body in the hind limbs was identified. For mild moxibustion, hairs within 1 cm × 2 cm of each acupoint were shaved to expose and disinfect the surface of the skin tissue using an alcohol-soaked cotton ball. A self-made moxibustion device (Figure 1(b)) was affixed to the rat, and 1 mm diameter of moxa stick (Nanyang Hanyi Moxa Co., Ltd., Henan, China; 120 mm in length, containing moxa wool, with combustion temperature of 40–60°C [18]) was applied 2 cm away from the skin after the rats had calmed down. Rats of the sham-operation group and model group were fastened on the operation bench as for the moxibustion groups every other day for a total of 12 weeks but moxibustion was not applied to these groups.

### 2.4. Specimen Processing and Detection

At the end of the 12-week intervention, all rats were sacrificed, and blood was collected from the abdominal aorta and centrifuged to prepare supernatant for the detection of serum creatinine, urea nitrogen, and uric acid. The right kidney of each animal was dissected out to remove the renal capsule and cut into two slices from the renal hilum, with one slice fixed in 10% neutral formalin solution for histopathology and immunohistochemistry and the other slice preserved in solution for subsequent RNA extraction and RT-PCR. One day before sacrifice, all animals were fasted in metabolic cages (Suzhou Fengshi Laboratory Animal Equipment Co., Ltd., Jiangsu, China) with free access of water, and urine samples were collected for 24 h, followed by centrifugation to measure the urinary protein levels.

The urinary protein level, serum creatinine, urea nitrogen, and serum uric acid were detected by the Beckman Coulter AU580 automatic biochemical analyzer in the biochemical laboratory of the Shanghai University of TCM. The assay kits were purchased from Beckman Coulter Experimental System Co. (Suzhou, China) and World Connaught Clinical Diagnostic Product Co., Ltd. (Japan). Expression of renal FN, *α*-SMA, and TGF-*β* was detected by immunohistochemistry and analyzed using Image-Pro Plus 6.0 image processing software. Antibodies for immunohistochemistry were purchased from Abcam (Cambridge, UK). Expression of renal podocin and nephrin was detected by Western blot analysis. Anti-podocin antibody was purchased from Abcam, and anti-nephrin antibody was purchased from Santa Cruz Biotechnology. Anti-GAPDH antibody was purchased from Cell Signaling Technology (Danvers, MA). Goat anti-rabbit, donkey anti-goat, and goat anti-mouse secondary antibodies were purchased from Beyotime Institute of Biotechnology (Shanghai, China). Renal podocin and nephrin mRNAs were measured by RT-PCR. The primers for PCR were synthesized by Shanghai Generay Biotech. Co., Ltd. (Shanghai, China; Table 1). The pathomorphology of the glomeruli and tubulointerstitium of the kidney tissues was observed under light microscopy (Olympus, Tokyo, Japan) after conventional dehydration, clearing, paraffin-embedding, sectioning into 3 *μ*m, and Masson staining.

### 2.5. Statistical Analysis

SPSS 17.0 software (SPSS Inc., Chicago, IL) was used for statistical analysis in this study. The normally distributed data were presented as means ± standard deviation ($\overset{-}{x}\pm s$). One-way ANOVA was used for comparison among groups. The least-significant difference (LSD) test was used to make comparisons between any two groups. The urinary protein was quantitatively analyzed by repeated measurement of variance. When *α* = 0.05, *P* < 0.05 was considered as an indication of statistical significance of the test data. *P* < 0.01 was considered extremely statistically significant.

## 3. Results

### 3.1. Establishment of a Rat FSGS Model

To establish a model for rat FSGS, we subjected rats to single nephrectomy followed by repeated injection of doxorubicin. The urinary protein levels for all FSGS rats were more than 100 mg/24 h, indicating FSGS model was successfully replicated. No rats died during the surgery or doxorubicin treatment, but 13 rats died after the FSGS modeling (29.55% mortality), mostly occurring in the 6th week after the second injection of doxorubicin. Anatomical analysis suggested that the deaths may be caused by doxorubicin-induced cardiotoxicity, adverse reactions in digestive system, and glomerular sclerosis. These results confirm the successful establishment of the FSGS model.

### 3.2. Effects of Moxibustion on Urinary Protein in the FSGS Rat Model

**Figure 2:**
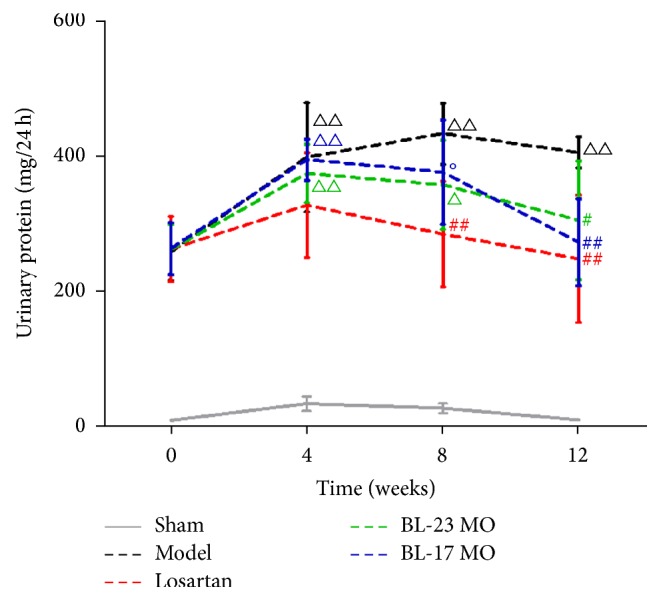
Moxibustion ameliorates proteinuria in FSGS rats. Data are expressed as the mean ± SD. Comparison between groups at the same time point: ^#^*P* < 0.05 and ^##^*P* < 0.01 versus model group; °*P* < 0.05 versus losartan group; ^△^*P* < 0.05 and ^△△^*P* < 0.01 versus week 0 within the group. MO, moxibustion.

To assess the effects of moxibustion on FSGS, we randomly divided the FSGS rats into four groups: the model group (no therapeutic treatment), losartan group (positive control therapy), Shenshu moxibustion group (BL-23 MO), and Geshu moxibustion group (BL-17 MO). The urinary protein levels in the four groups of FSGS rats were compared to each other and that of sham-operated rats (Figure 2 and Table 2). As expected, the urinary protein levels in the four FSGS groups were uniformly greater than in the sham group. Over time, the urinary protein levels increased in the model group, reaching a peak at 8 weeks and remaining high at 12 weeks. However, for the losartan, Shenshu moxibustion, and Geshu moxibustion groups, the urinary protein levels declined after week 4. Repeated measures ANOVA indicated that time and group factors significantly affected the urinary protein levels. Comparison over time demonstrated that the urinary protein levels within the FSGS model group were significantly higher at the end of the 4th, 8th, and 12th weeks than at week 0 (*P* < 0.05); however, for the losartan, BL-23MO, and BL-17 MO groups, the urinary protein content declined by week 12 to levels that were not significantly different than the levels at week 0 within each group. Comparison among groups indicated that, at the end of the 12-week intervention period, urinary protein levels of the losartan, Shenshu moxibustion, and Geshu moxibustion groups were significantly lower than those of the FSGS model group (*P* < 0.05, *P* < 0.01). No significant difference of the urinary protein level was observed between the losartan, Shenshu moxibustion, and Geshu moxibustion groups at the end of the 12-week intervention period (*P* > 0.05). Because urinary protein is a primary indication of kidney function, these results indicate that Shenshu moxibustion and Geshu moxibustion protected the rats from the damaging effects of FSGS on the kidneys.

### 3.3. Effects of Moxibustion on Kidney Function in the FSGS Rat Model

**Figure 3:**
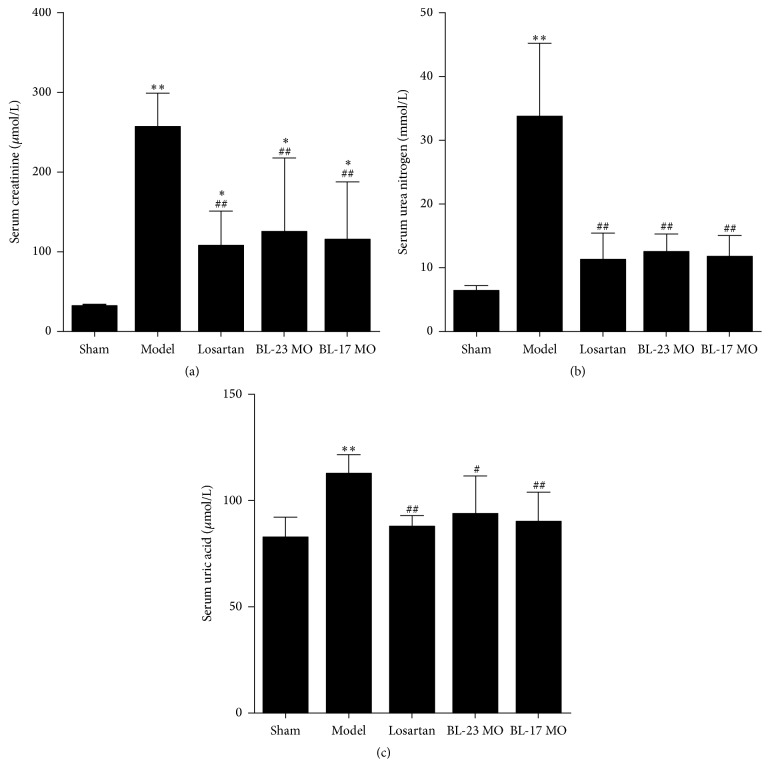
Moxibustion ameliorates renal dysfunction in FSGS rats. The levels of creatinine (a), urea nitrogen (b), and uric acid (c) in serum from different groups of rats. Data represent at least 6 rats per group and are expressed as the mean ± SD. ^*∗*^*P* < 0.05 and ^*∗∗*^*P* < 0.01 versus sham group. ^#^*P* < 0.05 and ^##^*P* < 0.01 versus model group.

To further evaluate the effects of moxibustion on kidney damage by FSGS, we measured serum markers in the different groups of rats at 12 weeks (Figure 3). Serum creatinine, urea nitrogen, and uric acid were significantly increased in the FSGS model group compared to the sham-operation group (*P* < 0.01). Additionally, serum creatinine levels were significantly increased in the losartan, Shenshu moxibustion, and Geshu moxibustion groups compared with the sham-operation group (*P* < 0.05); however, the increases relative to the sham group were less pronounced for these three treatment groups than for the FSGS model group (*P* < 0.05). Additionally, the serum urea nitrogen and uric acid contents were significantly reduced in the losartan, BL-23 MO, and BL-17 MO groups compared to the model group and were not significantly different from that of the sham group. These results are consistent with the results of the urinary protein analysis and provide additional evidence that moxibustion ameliorates kidney damage.

### 3.4. Effects of Moxibustion on FN, *α*-SMA, and TGF-*β* Expression in Kidney Tissues of FSGS Rats

**Figure 4:**
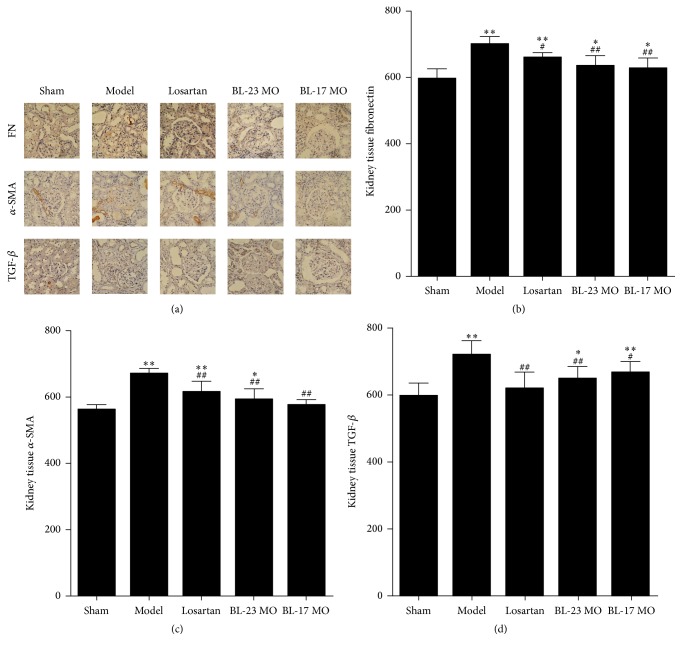
Moxibustion attenuates renal fibrotic lesions in vivo. Immunohistochemical staining of fibronectin (a), *α*-SMA (b), and TGF-*β* (c) in the kidneys. Data represent at least 6 rats per group and are expressed as the mean ± SD. ^*∗*^*P* < 0.05 and ^*∗∗*^*P* < 0.01 versus sham group. ^#^*P* < 0.05 and ^##^*P* < 0.01 versus model group.

To further examine the effects of moxibustion on kidney toxicity, we performed immunohistochemistry for biomolecules that are associated with renal disease (Figure 4). Compared with the sham-operation group, expression of renal FN, *α*-SMA, and TGF-*β* was significantly elevated in the FSGS model group and the Shenshu moxibustion group (*P* < 0.05, *P* < 0.01). Expression of renal FN and *α*-SMA was also significantly elevated in the losartan group (*P* < 0.01); and renal TGF-*β* expression was also elevated in the Geshu moxibustion group (*P* < 0.05, *P* < 0.01). Compared with the FSGS model group, the expression of renal FN, *α*-SMA, and TGF-*β* of the losartan group, the Shenshu moxibustion, and Geshu moxibustion groups was significantly reduced (*P* < 0.05, *P* < 0.01). Furthermore, compared with the losartan group, expression of renal FN and *α*-SMA of the Geshu moxibustion group was significantly reduced (*P* < 0.05, *P* < 0.01). These results are consistent with the ability of moxibustion to attenuate the effects of FSGS in rats.

### 3.5. Effects of Moxibustion on Renal Podocin and Nephrin mRNA and Protein Expression in FSGS Rats

**Figure 5:**
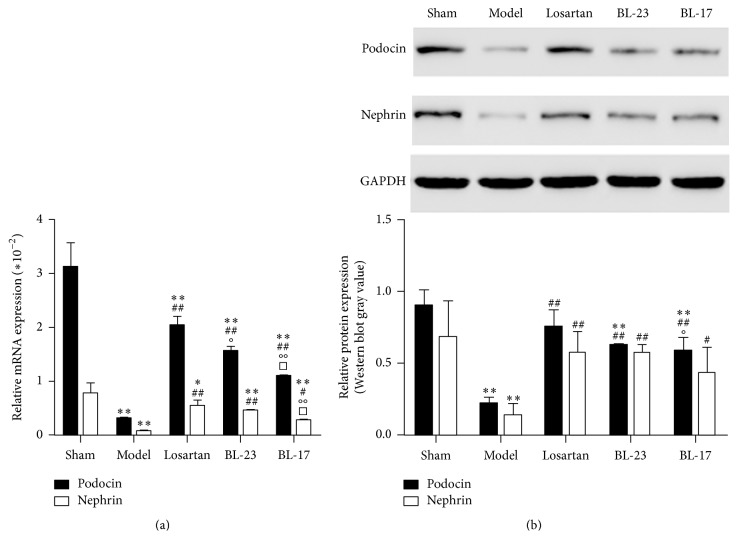
Moxibustion attenuates podocyte injury in vivo. (a) Real-time PCR analyses of podocin and nephrin in rat kidney tissues. (b) Western blot analyses of podocin and nephrin in the kidneys. Data represent groups of 3 rats and are expressed as the mean ± SD. ^*∗*^*P* < 0.05 and ^*∗∗*^*P* < 0.01 versus sham group. ^#^*P* < 0.05 and ^##^*P* < 0.01 versus model group. °*P* < 0.05 and °°*P* < 0.01 versus losartan group. ^□^*P* < 0.05 versus BL-23 group.

To further assess the effects of moxibustion on FSGS progression, we examined the mRNA and protein expression of podocin and nephrin (Figure 5), which are components of a complex that mediates kidney filtration [19]. Compared with expression in the sham-operation group, expression of renal podocin and nephrin mRNA was significantly reduced in the FSGS model group, the losartan group, the Shenshu moxibustion, and Geshu moxibustion groups (*P* < 0.05, *P* < 0.01); however, the reduction was less pronounced in the losartan, Shenshu moxibustion, and Geshu moxibustion groups compared to the model group (*P* < 0.05, *P* < 0.01). Similar results were obtained for podocin and nephrin protein. These results support the protective role of moxibustion on kidney filtration.

### 3.6. Effects of Moxibustion on the Renal Pathomorphology of FSGS Rats

**Figure 6:**
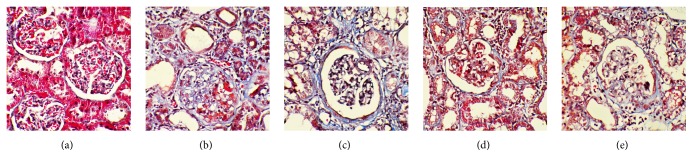
Moxibustion attenuates glomerular lesions in vivo. Representative micrographs demonstrate kidney injury at 12 weeks after treatment in different groups. Kidney sections were subjected to Masson's trichrome staining.

To directly examine the pathological effects of FSGS to the kidneys and the ability of moxibustion to attenuate these effects, we examined the histomorphology of kidney sections (Figure 6). In the sham-operation group (Figure 6(a)), the glomerular capillary loops were opened, and glomerular sclerosis was not observed; the renal tubular epithelial cells were arranged neatly; few fibroblasts were found in the interstitium, and inflammatory cells were only occasionally seen. In the rats of FSGS model group (Figure 6(b)), glomerular capillary loops were collapsed; some capillaries were narrow or even occluded; and segmental glomerulosclerosis was observed. In addition, tubular lesions were relatively severe, with swelling of tubular epithelial cells, granular degeneration, necrosis, loss of obvious tubular expansion, a large number of tubes, and some tubular atrophy; interstitial fibrosis and inflammatory cell infiltration were also obvious. Comparatively, the areas of the glomerular capillaries were shrunk in the rats of losartan group (Figure 6(c)), which had some narrowed capillaries and improved glomerular segmental sclerosis. Renal tubular epithelial cells in this group of rats were slightly swollen, showing interstitial fibrosis and small amount of inflammatory cell infiltration. Similarly, glomerular capillaries in the rats of the Shenshu moxibustion (Figure 6(d)) and Geshu moxibustion (Figure 6(e)) were partially narrowed, with mild glomerular segmental sclerosis, obvious interstitial fibrosis, and small amount of inflammatory cell infiltration. These results provide morphological evidence for the protective effects of moxibustion.

## 4. Discussion

In this study, we assessed the ability of moxibustion monotherapy to intervene with disease progression in the FSGS rat model and monitored its impacts on urinary protein levels, renal function, and relevant biomolecules. Our results demonstrate that the 24-h urinary protein, serum creatinine, urea nitrogen, and serum uric acid of FSGS rats were improved to certain degrees after administration of two different moxibustion protocols (MO-BL-23 and MO-BL-17). In addition, the renal expression of FN, *α*-SMA, TGF-*β*, podocin, and nephrin and the pathomorphology of the kidney tissues also showed positive changes after moxibustion therapy.

To evaluate the effects of moxibustion, we first examined its effects on urinary protein levels, which is a common indicator of kidney failure. We selected losartan as a positive control therapy in these experiments because it is used in patients with primary FSGS to delay progression of chronic kidney disease to end stage kidney disease when corticosteroids and immunosuppressive therapy fail to induce remission [20]. By blocking the renin-angiotensin-aldosterone system, losartan provides regulation of tubulointerstitial injury and fibrosis to improve the glomerular filtration rate, reduce tubulointerstitial injury and fibrosis, and enable renal protection. The urinary protein levels of the FSGS rats were significantly higher than those of the sham-operation group at all time points, regardless of therapeutic application. Furthermore, the protein showed a time-dependent pattern, with continued elevation on weeks 0, 4, and 8 and a slight drop on week 12 in the model group. In contrast, the 24-h urinary protein levels of the losartan group, the Shenshu moxibustion, and the Geshu moxibustion groups showed a pattern of reduction over time. While the therapeutic effects in this assay were most obvious for losartan, these results suggest that moxibustion may also have therapeutic potential. Given that moxibustion is applied externally and functions by enhancing a natural system within the body, it could provide a less toxic and less invasive alternative therapy with similar effect to oral or injectable pharmaceuticals.

The production of urinary proteins is inseparable from podocyte injury [21], and elevation of urinary protein further enhances the severity of podocyte injury in turn, resulting in the progression of glomerular sclerosis. This study showed that expression of renal podocin and nephrin mRNA in FSGS rats of different treatment groups was significantly lower than that of the sham group. These results confirm that the renal toxicity of doxorubicin caused glomerular podocyte injury and reduced podocyte marker protein expression, which led to podocyte loss or apoptosis. The expression of renal podocin and nephrin protein and mRNA of the losartan Shenshu moxibustion and Geshu moxibustion groups was significantly higher than that of the FSGS model group, suggesting that moxibustion alleviates the shedding and missing of podocyte marker proteins in kidney tissues after FSGS. Thus, the relevant mechanism of moxibustion in reducing the elevation in urinary protein content after FSGS induction may involve an increase in renal podocin and nephrin protein expression, thereby maintaining the structural integrity of podocyte septa and subsequently alleviating podocyte injury.

We also demonstrated that the serum creatinine, urea nitrogen, and uric acid of the losartan, Shenshu moxibustion, and Geshu moxibustion groups were significantly higher than those of the sham-operation group but lower than those of the FSGS model group, suggesting that FSGS rats had different degrees of renal dysfunction after therapeutic application. In addition, these three indicators had no significant difference in different treatment groups as assessed by pairwise comparison, suggesting that Shenshu moxibustion and Geshu moxibustion significantly improve the renal function of FSGS rats, with similar effects to losartan treatment.

The degree of renal interstitial fibrosis is closely associated with the renal function. Doxorubicin nephrotoxicity and stimulation of excessive proteinuria induce the release of a variety of cytokines and inflammatory mediators produced by glomerular intrinsic cells and other inflammatory cells to stimulate the proliferation of glomerular mesangial cells and increase of the mesangial matrix, alleviating the development and formation of glomerulosclerosis [22]. In addition, by inducing interstitial inflammatory cell infiltration, doxorubicin nephrotoxicity, and proteinuria, renal tubular injury can lead to interstitial fibrosis, ultimately causing renal fibrosis, which could progress to end stage renal failure. In this study, we demonstrated that expression of renal *α*-SMA, TGF-*β*, and FN in rats of the FSGS model group, losartan group, Shenshu moxibustion group, and Geshu moxibustion group was higher than in the sham-operation group, suggesting that renal interstitial fibrosis occurs in rats after FSGS modeling. In the Shenshu moxibustion and Geshu moxibustion groups, the above indexes were relatively lower than in the FSGS model group, while expression of renal *α*-SMA and FN of the Geshu moxibustion group was lower than the losartan group, suggesting that Shenshu moxibustion, Geshu moxibustion, and losartan treatment improve the renal interstitial fibrosis of FSGS rats. The regulatory effect of moxibustion on renal tubular epithelial-mesenchymal transition and extracellular matrix (ECM) integrity in FSGS rats was similar for the losartan treatment. Therefore, according to this measure, the inhibitory effect of *α*-SMA and FN expression by Geshu moxibustion may be more significant than by losartan.

Podocytes are the major cells participating in the development of FSGS. Denaturation occurs after podocyte injury and the separation of glomerular basement membrane (shedding) causes expansion of the capillary loop, exposure of the tubular basement membrane, and Bowman capsule adhesion. Through tearing, stretching, and other changes, gradual progression of the disease occurs to the entire glomerulus and its connected tubules. In addition, ECM production of the focal lesion continuously increases and is characterized by compression of the capillary loop occlusion, which eventually leads to the formation of glomerular sclerosis. In this study, doxorubicin-induced FSGS modeling in different groups of rats caused different degrees of glomerular structural disorder, collapse, and atrophy, and some of the rats showed glomerular sclerosis. Pathological injury of rat kidney tissues in the losartan, Shenshu moxibustion, and Geshu moxibustion groups was relatively less severe compared to the FSGS model group, which is consistent with the pattern of expression of renal fibrosis- and podocyte injury-related markers. These findings confirm that podocyte injury and ECM accumulation (renal interstitial fibrosis) are closely associated with the incidence of glomerular sclerosis in our model.

Moxibustion has been extensively applied, but a lack of sufficient data is available to demonstrate its effectiveness in the scientific research field. Most previous studies have focused on randomized controlled trials of acupuncture combined with moxibustion; however, moxibustion monotherapy is rarely reported. Our study evaluated the effects of moxibustion without acupuncture and compared the acupoint compatibilities of moxibustion monotherapy for the BL-23 and BL-17 acupoints. The BL-23 acupoint has been more frequently used in studies of chronic kidney diseases [6–8], while BL-17 acupoint has been reported in the treatment of a variety of diseases [23–25]. Randomized controlled trials that have evaluated these acupoints involved a large number and different combinations of acupoints, which has made it difficult to accurately determine the role of single-acupoint intervention for specific acupoint.

As a limitation, this study did not include nonspecific acupoint moxibustion control groups for rats without FSGS. However, the results affirm the therapeutic effect of the moxibustion in FSGS. Additionally, because no statistically significant differences were found between the two-moxibustion groups in this study, we could not validate the specific therapeutic effect of moxibustion at the two acupoints. Studies that assess the efficacy of moxibustion in ameliorating proteinuria that results from chronic kidney diseases are rare, as is acupoint-specificity-related research. Therefore, our work provides a unique cross-section of a field that is in need of further investigation. Future studies will be based on supporting the current findings to improve the experimental design and conduct further in-depth study.

## 5. Conclusions

In this study, we applied single nephrectomy and repeated injection of doxorubicin to a FSGS model in rats and used moxibustion at Shenshu and Geshu acupoints to confirm that the moxibustion improved urinary protein levels and renal function. Shenshu and Geshu moxibustion also alleviated podocyte injury and inhibited renal interstitial fibrosis, thereby improving the glomerulosclerosis.

## Figures and Tables

**Table 1:** Primer sequences and RT-PCR condition.

Gene	Primer sequence		Size (bp)	Product GC (%)
Podocin	Primer F	5′ ACCCAAGTTCTCCCAAACC 3′	226	50
	Primer R	5′ CTGCCATTTCACTGCGTTC 3′		
Nephrin	Primer F	5′ AGAGACTGGGAGAAGAAGAG 3′	185	57
	Primer R	5′ AGCAAATCGGACGACAAG 3′		
GAPDH	Primer F	5′ GTCGGTGTGAACGGATTTG 3′	181	51
	Primer R	5′ TCCCATTCTCAGCCTTGAC 3′		

**Table 2:** Comparison of 24-h urinary protein in each group of rats (mean ± standard deviation, mg/24 h).

Group	*n*	0th week	4th week	8th week	12th week
Sham	7	11.19 ± 3.34	35.60 ± 10.70	28.83 ± 7.05	11.70 ± 3.90
Model	6	258.32 ± 42.41	397.17 ± 79.97^△△^	431.73 ± 44.99^△△^	404.15 ± 22.77^△△^
Losartan	6	262.28 ± 47.75	326.81 ± 77.13	284.23 ± 77.40^##^	248.09 ± 93.67^##^
BL-23 MO	6	261.64 ± 36.78	373.16 ± 42.87^△△^	356.50 ± 65.62^△^	304.36 ± 87.14^#^
BL-17 MO	6	262.99 ± 38.04	393.53 ± 30.08^△△^	375.16 ± 76.51°	272.27 ± 63.39^##^

^#^ *P* < 0.05 and ^##^*P* < 0.01 versus model group; °*P* < 0.05 versus losartan group; ^△^*P* < 0.05 and ^△△^*P* < 0.01 versus week 0 within the group.
